# Dissecting sexual minority subgroup differences in the association between depression, anxiety, and cognitive difficulties

**DOI:** 10.1371/journal.pone.0295821

**Published:** 2024-01-03

**Authors:** Ethan Morgan, Christina Dyar, Karen Rose

**Affiliations:** 1 College of Nursing, The Ohio State University, Columbus, OH, United States of America; 2 College of Public Health, The Ohio State University, Columbus, OH, United States of America; Northumbria University, UNITED KINGDOM

## Abstract

**Introduction:**

A growing body of research has demonstrated extensive mental health disparities affecting sexual minority populations, yet little research has assessed how these disparities may affect cognitive functioning among subgroups of sexual minorities.

**Methods:**

Data come from the 2021 National Health Information Survey (NHIS). Survey-weighted linear regression analyses were used to assess self-reported measures of cognition, stratified by subgroups sexual identity. In particular, we focused on the association between symptoms of depression or anxiety and each of the measures of cognition, adjusting for demographic covariates.

**Results:**

Among 31,994 NHIS participants in the 2021 survey, 5,658 (17.7%) reported at least some difficulty in remembering or concentrating. Basic demographic differences existed when assessing any cognitive difficulty, particularly for bisexual participants (aOR = 2.73; 95% CI: 2.07, 3.60) and participants identifying as a different identity (aOR = 4.22; 95% CI: 2.72, 6.56). Depression was significantly associated with cognitive difficulty with the largest relationship observed among gay/lesbian participants (aOR = 1.39; 95% CI: 1.29, 1.49). The association between anxiety and cognitive difficulty was smallest among bisexuals (aOR = 1.13; 95% CI: 1.08, 1.18) and relatively consistent across other subgroups: heterosexuals (aOR = 1.23; 95% CI: 1.22, 1.24), gay/lesbians (aOR = 1.27; 95% CI: 1.19, 1.36), and those with a different identity (aOR = 1.22; 95% CI: 1.10, 1.35).

**Conclusion:**

There is a clear set of health disparities between sexual minority subgroups and heterosexuals across all cognitive difficulties. Future research should focus on developing a better understanding of differences in cognition based on sexual minority status while also working to ascertain how disparities vary among sexual minorities.

## Introduction

A growing body of research has demonstrated extensive health disparities affecting sexual minority populations, relative to their heterosexual counterparts [1–3]. For example, sexual minorities are at increased risk for both physical health disparities (e.g., stroke, cardiovascular disease, cancer, and diabetes [4–7]) and mental health disparities (e.g., depression and anxiety [8–10]). A key driver of these disparities can be found in stigma-related stressors (i.e., chronic stress arising from the stigmatization of non-heterosexuality) [11, 12]. Minority stress theory posits that chronic exposure to stigma-related stressors contributes to health disparities affecting sexual minorities by depleting coping resources, leading to adverse health outcomes, including substance use, anxiety, depression, and physical health conditions [13–16]. Even in light of recent increases in health disparity research among sexual minorities, little work has focused on differences in cognitive difficulties between sexual minority and heterosexual populations.

Cognitive difficulties are not caused by any one disease or condition and range from very mild (e.g., more memory loss than others of a similar age) to quite severe (e.g., inability to function outside of assisted living facilities). They can broadly be described as anytime someone has difficulty remembering (e.g., past events, daily activities), difficulty concentrating on tasks, trouble learning new skills or tasks, not recognizing faces of known people, memory loss, etc. [17]. One potentially key risk factor for cognitive difficulty and decline among sexual minority populations is their consistently higher rates of both depression and anxiety relative to heterosexuals [18]. These findings are likely due to a set of intersecting identities among older sexual minority populations and their risk of exposure to multiple forms of discrimination. For example, not only do they experience sexual orientation-related stigma but they also experience higher rates of age-related discrimination (e.g., discrimination in long-term care facilities) [10, 19–22]. Recent work has taken these findings a step further by demonstrating that sexual minorities who report more aging-related concerns also report higher rates of both depression and anxiety [10]. And although these mental health factors are known to increase risk for cognitive decline and impairment [23–26], little work has assessed their association with cognitive functioning among sexual minority populations, let alone subgroup differences (e.g., gay/lesbian versus bisexuals).

Initial studies have demonstrated that up to a third of sexual minority samples report difficulty with memory or at least one of six cognitive functions [27, 28], compared to approximately 11% among the general population [29]. More recent work has expanded on these earlier studies by using data from a nationally representative population, the National Social Life, Health, and Aging Project, noting that sexual minority older adults (≥50 years) had significantly greater cognitive impairment than heterosexual older adults [30]. Unfortunately, as is the issue with many national studies, there was insufficient sample size in this study to assess subgroups of sexual minorities (e.g., gay/lesbian, bisexuals) nor to fully assess other mechanisms driving disparities in cognition. This is a common theme making it difficult to properly assess extant health disparities among sexual minorities. For example, the National Health and Nutrition Examination Survey last asked sexual identity and cognition questions in the same survey in 2013–2014, data that is now nearly a decade old and does not adequately assess cognition nor does it include measures which assess gender identity. Studies that do have larger samples of sexual minorities, such as the Youth Risk Behavior Surveillance System, focus on younger populations which limits their utility in understanding chronic disease health disparities.

To begin to address these gaps in the literature, we used data from the National Health Information Survey to assess the relationship between sexual identity and individual, self-reported assessments of cognition. We also aimed to determine the extent to which this relationship is confounded by depression and anxiety severity symptoms. Secondarily, we assessed differences in the relationship between depression and anxiety symptoms and cognition by stratifying the analyses by sexual identity.

## Methods

### Study population

Data in this analysis come from the 2021 National Health Interview Survey (NHIS) dataset, a publicly available, nationally representative cross-sectional survey of civilian non-institutionalized U.S. citizens residing in the 50 states or the District of Columbia at the time of interview [31]. This is an annual survey that provides information on a host of health-related factors, specializing in categorization by demographic and socioeconomic characteristics. Survey sample weights are calculated and provided by NHIS as part of the publicly available data and are used to obtain nonbiased estimates for survey outcomes. Only data from the 2021 NHIS survey are utilized here as the survey composition and weighting scheme underwent major change in 2019 and 2020 data were heavily influenced by the COVID-19 pandemic. All data are deidentified and publicly available, thus are exempted from Institutional Review Board review.

### Demographic measures

Survey participants self-reported their demographic information: age, sex, sexual identity, race and ethnicity, and current health insurance status. Age was operationalized as a continuous variable (range 18–99 years). Sex was assessed as a dichotomous variable, female or male. NHIS does not assess gender identity. Race and ethnicity were provided in the dataset and categorized by NHIS as: Hispanic, non-Hispanic white only, non-Hispanic Black only, non-Hispanic Asian only, non-Hispanic Native American/Alaska Native only, non-Hispanic Native American/Alaska Native and any other race, and any different race or multiracial. Participants reporting a Hispanic ethnicity were coded as such, regardless of their racial identity. Sexual identity was assessed in the survey as, “Do you think of yourself as gay/lesbian; straight, that is, not gay/lesbian; bisexual; something else; or you don’t know the answer?”, and coded as lesbian/gay, heterosexual, bisexual, or a different identity. Lastly, possession of any health insurance at time of interview (e.g., private insurance, Medicare, Medicaid, Tricare, etc.) was operationalized as a dichotomous variable.

### Cognition

In NHIS, cognition was assessed by examining any difficulty remembering or concentrating, frequency of this difficulty, and amount of things with which one has difficulty. The first of these was asked as, “Do you have difficulty remembering or concentrating?”, operationalized as a categorical variable: no difficulty, some difficulty, a lot of difficulty, and cannot do at all (referred to as cognitive difficulty throughout). Second, among those who have difficulty remembering or concentrating, “How often do you have difficulty remembering? Would you say sometimes, often, or all of the time?”, categorized as asked in the question (referred to as frequency of difficulty throughout). And third, again, among those who have difficulty remembering or concentrating, “Do you have difficulty remembering a few things, a lot of things, or almost everything?” and categorized as such (referred to as amount of difficulty throughout).

### Mental health

Depression symptom severity was assessed using the Patient Health Questionnaire 8 (PHQ-8) [32, 33]. Often used in place of the PHQ-9, the PHQ-8 eliminates the final question regarding thoughts about death and self-harm, it is typically eliminated as it does not assess suicidality and positive answers on this question do not correlate with suidality. The PHQ-8 is scored on a scale ranging from 0–24 with larger values indicating greater depression symptom severity, the range reported in the data among all participants was 0–24. Anxiety symptom severity was assessed using the Generalized Anxiety Disorder 7 (GAD-7) [34]. The GAD-7 is scored on a scale ranging from 0–21 with larger values indicating greater anxiety symptom severity, the range reported in the data among all participants was 0–21. Both measures were utilized as continuous variables to examine their association with each of the measures of cognition, particularly when stratified by sexual identity.

### Statistical analyses

Participant characteristics were described using means, standard deviations, and proportions, as appropriate. Survey-weighted multivariable ordinal regression models were first utilized to assess the association between sexual identity and each of the cognitive difficulties as the key outcomes, adjusting for demographic characteristics and insurance status. Next, we examined differences in the association between depression or anxiety symptoms and each of the cognitive difficulties by stratifying by sexual identity. All models were weighted using only the individual survey weights provided by NHIS; primary sampling units (PSU) and strata were not included as sexual identity categories belonged to only one PSU, thus standard errors and confidence intervals could not be calculated. Data were not additionally stratified by sex as there was insufficient power to stratify models by both sex and sexual identity. Statistical significance was established at alpha <0.05. All analyses were performed in Stata 17.0.

## Results

Among the 31,994 NHIS participants during the study period (Table 1), mean difficulty remembering or concentrating was 1.20 (SD = 0.46), frequency of this difficulty was 1.38 (SD = 0.70), and amount of things with which one has difficulty was 1.24 (SD = 0.55). This indicates that, on average, most participants have no to some difficulty remembering or concentrating. And among those reporting any difficulty, the frequency varies from sometimes to often while the amount of things with which one has difficulty varies from a few to a lot of things. Stratifying these by sexual identity, those identifying as a different sexual identity reported the greatest difficulty remembering or concentrating (mean = 1.39, SD = 0.58), followed by bisexuals (mean = 1.34, SD = 0.57), heterosexuals (mean = 1.20, SD = 0.45), and gay/lesbians (mean = 1.19, SD = 0.45). Frequency of difficulty remembering or concentrating was highest among bisexuals (mean = 1.55, SD = 0.70), followed by those identifying as a different sexual identity (mean = 1.51, SD = 0.78), gay/lesbians (mean = 1.44, SD = 0.70), and heterosexuals (mean = 1.37, SD = 0.65). Amount of difficulty was greatest among those identifying as a different sexual identity (mean = 1.38, SD = 0.68), followed by gay/lesbians (mean = 1.30, SD = 0.63), bisexuals (mean = 1.26, SD = 0.56), and heterosexuals (mean = 1.23, SD = 0.53).

**Table 1:** Demographic characteristics of participants in the analytic sample, NHIS, 2021 (N = 31,994).

Variable	N	%	Mean	SD
**Age**	–	–	52.1	18.5
**Sex** ^**1**^				
Female	17,261	54.0	–	–
Male	14,733	46.1	–	–
**Sexual Identity**				
Heterosexual	29,818	96.8	–	–
Lesbian/Gay	452	1.5	–	–
Bisexual	413	1.3	–	–
Different Identity	122	0.4	–	–
**Race**				
Hispanic	4,152	13.0	–	–
NH White only	21,915	68.5	–	–
NH Black only	3,483	10.9	–	–
NH Asian only	1,648	5.2	–	–
NH AIAN only	212	0.7	–	–
NH AIAN and any other group	248	0.8	–	–
Other single or multiple race(s)	339	1.1	–	–
**Health Insurance, any**	29,655	92.8		
**Mental Health**				
*PHQ-8 Depression Score*	–	–	2.5	4.0
*GAD-7 Anxiety Score*	–	–	2.0	3.8
**Cognitive Functioning**				
Cognitive Difficulty	–	–	1.2	0.5
Frequency of Difficulty	–	–	1.4	0.7
Amount of Difficulty	–	–	1.2	0.5

Abbreviations: NHIS = National Health Interview Survey; SD = standard deviation; NH = Non-Hispanic; AIAN = American Indian/Alaska Native.

^1^Percentages total slightly over one hundred percent due to rounding

Mean age was 52.1 years (Standard Deviation [SD] = 18.5). The majority of participants self-identified as female (n = 17,261, 54.0%), heterosexual (n = 29,818, 96.8%), and had insurance at the time of the interview (n = 29,655, 92.8%). Regarding race and ethnicity, 21,915 (68.5%) identified as non-Hispanic White only, 4,152 (13.0%) as Hispanic, 3,483 (10.9%) as non-Hispanic Black only, 1,648 (5.2%) as non-Hispanic Asian only, 339 (1.1%) as any other race or multiple race(s), 248 (0.8%) as non-Hispanic American Indian or Alaska Native and any other race, and 212 (0.7%) as non-Hispanic American Indian or Alaska Native only. The mean PHQ-8 depression symptom score was 2.5 (SD = 4.0) and the mean GAD-7 anxiety symptom score was 2.0 (SD = 3.8), both indicating none-to-minimal depressive or anxious symptoms.

Table 2 presents multivariable survey-weighted ordinal logistic regression analyses examining the association between demographic characteristics and each of the three cognitive difficulties. The first examined overall cognitive difficulty. In this model, compared to heterosexuals, bisexual participants (adjusted odds ratio [aOR] = 2.73; 95% confidence interval [CI]: 2.07, 3.60) and those identifying as a different identity (aOR = 4.22; 95% CI: 2.72, 6.56) self-reported greater cognitive difficulty. Age was also associated with greater cognitive difficulty (aOR = 1.02; 95% CI: 1.02, 1.03) such that each year increase in age was associated with worse cognitive functioning. Compared to males, females reported greater cognitive difficulty. (aOR = 1.20; 95% CI: 1.11, 1.29). Regarding race and ethnicity, compared to non-Hispanic White only participants, Hispanic (aOR = 0.82; 95% CI: 0.72, 0.93) and non-Hispanic Asian only participants (aOR = 0.41; 95% CI: 0.32, 0.51) each exhibited less cognitive difficulties while those identifying as non-Hispanic American Indian and Alaska Natives along with any other race group (aOR = 2.76; 95% CI: 1.88, 4.04) exhibited more cognitive difficulties. And compared to those with no current form of insurance at the time of the interview, those with insurance had fewer cognitive difficulties (aOR = 0.85; 95% CI: 0.73, 1.00).

**Table 2:** Multivariable survey-weighted linear regression analyses examining the association between demographic characteristics and measures of cognitive difficulty, NHIS, 2021.

	Cognitive Difficulty		Frequency of Difficulty		Amount of Difficulty	
	aOR	95% CI	aOR	95% CI	aOR	95% CI
**Age**	1.02***	1.02, 1.03	1.00	0.99, 1.00	1.00	1.00, 1.01
**Race and Ethnicity**						
NH White Only	Ref	–	Ref	–	Ref	–
Hispanic	0.82**	0.72, 0.93	1.12	0.86, 1.46	1.47**	1.10, 1.96
NH Black Only	1.02	0.90, 1.15	1.03	0.80, 1.33	1.22	0.92, 1.62
NH Asian Only	0.41***	0.32, 0.51	0.65	0.40, 1.07	1.00	0.58, 1.72
NH AIAN Only	1.51*	1.00, 2.27	0.82	0.36, 1.84	1.04	0.43, 2.56
NH AIAN and any other group	2.76***	1.88, 4.04	1.68	0.95, 2.97	2.50*	1.10, 3.43
Different or multiple races	1.03	0.73, 1.46	1.39	0.66, 2.92	1.65	0.80, 3.43
**Sex**						
Male	Ref	–	Ref	–	Ref	–
Female	1.20***	1.11, 1.29	0.97	0.82, 1.13	0.95	0.79, 1.15
**Insurance Status**						
No Insurance	Ref	–	Ref	–	Ref	–
Any form of insurance	0.85*	0.73, 1.00	1.06	0.78, 1.45	0.85	0.60, 1.21
**Sexual Identity**						
Heterosexual	Ref	–	Ref	–	Ref	–
Gay/Lesbian	1.27	0.94, 1.71	1.35	0.70, 2.62	1.15	0.58, 2.29
Bisexual	2.73***	2.07, 3.60	1.96**	1.32, 2.91	1.50	0.85, 2.66
Different Identity	4.22***	2.72, 6.56	0.93	0.36, 2.42	1.05	0.40, 2.80

***p<0.001

**p<0.01

*p<0.05

Abbreviations: NHIS = National Health Information Survey; AIAN = American Indian/Alaska Native

^1^Models adjusted for age, sex, race/ethnicity, and health insurance status

Next, among those exhibiting any cognitive difficulty, we examined both frequency and amount of difficulty remembering. Regarding frequency of difficulty, only bisexual participants (aOR = 1.96; 95% CI: 1.32, 2.91) exhibited a higher frequency of difficulty remembering relative to heterosexual participants. Meanwhile, there was no significant difference in amount of difficulty remembering based on sexual identity.

Table 3 presents multivariable survey-weighted ordinal logistic regression analyses examining the association between each of the cognitive difficulties and symptoms of depression or anxiety, stratified by sexual identity. Here, across all sexual identities, there was a significant association between symptoms of depression and any difficulty concentrating or remembering such that a greater number of symptoms was associated with greater difficulty. The effect size was greatest among gay and lesbian participants (aOR = 0.07; 95% CI: 0.05, 0.08), followed by those with a different identity (aOR = 1.28, 95% CI: 1.5, 1.41), heterosexuals (aOR = 1.27; 95% CI: 1.25, 1.28), and bisexuals (aOR = 1.16; 95% CI: 1.10, 1.23. Similar findings were observed with regards to symptoms of anxiety: gay/lesbians (aOR = 1.27; 95% CI: 1.19, 1.36), heterosexuals (aOR = 1.23; 95% CI: 1.22, 1.24), those with a different identity (aOR = 1.22; 95% CI: 1.10, 1.35), and bisexuals (aOR = 1.13; 95% CI: 1.08, 1.18).

**Table 3:** Multivariable survey-weighted linear regression analyses examining the association between cognition, PHQ-8 depression, and GAD-7 anxiety, stratified by sexual identity, NHIS, 2021.

	Heterosexual^1^		Gay/Lesbian^1^		Bisexual^1^		Different Identity^1^	
	aOR	95% CI	aOR	95% CI	aOR	95% CI	aOR	95% CI
**Any Difficulty Concentrating or Remembering** ^**2**^								
*Depression*	1.27***	1.25, 1.28	1.39***	1.29, 1.49	1.16***	1.10, 1.23	1.28***	1.15, 1.41
*Anxiety*	1.23***	1.22, 1.24	1.27***	1.19, 1.36	1.13***	1.08, 1.18	1.22***	1.10, 1.35
**Frequency of Difficulty Remembering** ^**2,3**^								
*Depression*	1.11***	1.10, 1.13	1.04	0.91, 1.18	1.07	1.00, 1.16	–^4^	–
*Anxiety*	1.10***	1.08, 1.11	1.03	0.92, 1.16	1.11*	1.01, 1.21	–^4^	–
**Amount of Difficulty Remembering** ^**2,3**^								
*Depression*	1.14***	1.12, 1.16	1.43***	1.19, 1.73	1.06	0.96, 1.17	–^4^	–
*Anxiety*	1.12***	1.10, 1.14	1.43***	1.22, 1.68	1.04	0.93, 1.16	–^4^	–

***p<0.001

**p<0.01

*p<0.05

^1^Models adjusted for age, sex, race/ethnicity, and health insurance status

^2^Symptoms of depression and anxiety were assessed in separate models

^3^Among those with any difficulty concentrating or remembering

^4^Convergence not achieved

Among those who reported cognitive difficulty in concentrating or remembering, we subsequently examined similar models as above to assess frequency of difficulty remembering. Here, with regards to symptoms of depression, results held among heterosexuals (aOR = 1.11; 95% CI: 1.10, 1.13), but not gay/lesbian or bisexual participants, such that a greater number of symptoms of depression were associated with greater frequency of difficulty remembering. Meanwhile, regarding anxiety, results held among heterosexuals (aOR = 1.10; 95% CI: 1.08, 1.11) and bisexuals (aOR = 1.11; 95% CI: 1.01, 1.21), but not among gay/lesbian participants. Models did not converge among those with a different identity due to cell sizes.

Next, we examined associations between symptoms of depression or anxiety and the amount of difficulty remembering, again, among those who initially reported any difficulty in concentrating or remembering. In this set of models, a greater number of symptoms of depression were associated with a higher amount of difficulty remembering among heterosexuals (aOR = 1.14; 95% CI: 1.12, 1.16) and gay/lesbians (aOR = 1.43; 95% CI: 1.19, 1.73), but not among bisexuals. The same was true for symptoms of anxiety among heterosexuals (aOR = 1.12; 95% CI: 1.10, 1.14) and gay/lesbians (aOR = 1.43; 95% CI: 1.22, 1.68). Again, models did not converge among those with a different identity due to cell sizes.

## Discussion

In this nationally representative dataset, we examined three components of cognition and their relationships with sexual identity. Across several models, we noted varying relationships between measures of cognitive difficulties and self-reported symptoms of depression or anxiety based on sexual identity. Generally, both depression and anxiety were associated with worse cognitive functioning across sexual identities, however, these results were not always consistent even within specific sexual minority groups. For example, gay/lesbian participants who reported a greater number of symptoms of depression or anxiety had a more frequent difficulty remembering while bisexuals reported a higher amount of difficulty remembering. Results among those identifying with a different identity were more mixed. These results reinforce the notion that sexual minority populations are not homogeneous and more research on cognition among these populations, particularly subgroups of sexual minorities, is needed to reduce health disparities and to address the specific needs of each subgroup.

Consistent with recent work from the National Social Life, Health, and Aging project (NSHAP) [30], depressive symptoms explain a large portion of the variability in the relationship between self-reported cognition and sexual identity among the 2021 sample of NHIS participants. Our work expands on these past findings, however, by assessing subgroups of sexual minorities which NSHAP was not well powered to examine. Interestingly, we observed that, even after adjusting for either depression or anxiety symptoms, only those identifying as bisexual continued to report increased frequency in difficulty of remembering things. This is another data point in an expanding body of research demonstrating poor health outcomes specifically among bisexuals. To name a few, bisexual females face increased risk for physical health conditions as a result of the unique experiences of stigma [35–38]. And bisexual Black and Hispanic men experience more stigma related to HIV-preventing pre-exposure prophylaxis use, resulting in greater rates of discontinuation [39]. While all sexual minorities can experience minority stress, bisexuals experience unique forms that gay/lesbian individuals do not, such as denial and invalidation (e.g., the belief that bisexuality is not a legitimate sexual orientation) and erasure (e.g., assumptions of heterosexuality or homosexuality based on the gender of one’s partner) [40]. Further, bisexuals experience discrimination from both heterosexuals and other sexual minority groups, reducing their access to support from the broader community [35, 40]. Thus, it is vital that future studies on cognition continue to expand on this work by incorporating sufficient sample sizes to assess sexual minority subgroup differences.

Our work also goes beyond past studies by assessing differences by sexual identity in the relationship between depression or anxiety symptoms and measures of cognitive difficulties. In particular, regardless of sexual identity, there remains a significant relationship between depression or anxiety and having difficulty in concentrating or remembering. However, we demonstrated that the strength of this relationship differs by sexual identity. For example, these associations are consistently stronger among gay and lesbian participants relative to heterosexuals but then also consistently lower among bisexuals. But when examining frequency of difficult in remembering things the results remained significant only among bisexuals and heterosexuals, not gay and lesbian participants. These findings may be attributable to the aforementioned stressors that are specific to the bisexual population, suggesting yet another health disparity that may affect bisexuals. It is vital that future research focus on reducing health disparities assess differences by sexual minority subgroups and refrain from combining all sexual minorities into a single homogenous group as this may mask key differences.

### Limitations

Our study has several limitations that should be considered when interpreting our results. Although NHIS is a representative survey it relies on self-reported measures of cognitive difficulties and does not assess standard measures included in most studies focused on cognition. NHIS also excludes incarcerated and homeless individuals from participation in the survey, a study design that may inadvertently reduce the representation of sexual minorities given their higher likelihood of experiencing homelessness [41]. Next, the repeated cross-sectional design of NHIS limits our ability to assess causality, thus one ought to be careful to consider potential bidirectionality of some associations examined in this study (e.g., depression and cognitive difficulties). NHIS also does not assess gender identity, instead assessing only sex, preventing the examination of these relationships among transgender and non-binary populations. Finally, these measures of cognition are not ideal as they are more simplistic in nature and do not adequately assess the many facets of cognitive functioning.

### Clinical significance

Even considering these limitations, we observed several key findings. First, there is a clear set of health disparities between sexual minority subgroups and heterosexuals across all cognitive difficulties, suggesting that while sexual minorities are at risk of poor cognitive outcomes this risk differs based on their sexual identity. Second, a considerable portion of the relationship between sexual identity and cognition is explained by depression and anxiety but disparities in cognition persist for bisexuals. Future research should focus on developing a better understanding of differences in cognition based on sexual minority status while also working to ascertain how disparities vary among sexual minorities.
